# LiDAR-Based Non-Cooperative Tumbling Spacecraft Pose Tracking by Fusing Depth Maps and Point Clouds

**DOI:** 10.3390/s18103432

**Published:** 2018-10-12

**Authors:** Gaopeng Zhao, Sixiong Xu, Yuming Bo

**Affiliations:** School of Automation, Nanjing University of Science and Technology, Nanjing 210094, China; 116110001165@njust.edu.cn (S.X.); byming@njust.edu.cn (Y.B.)

**Keywords:** non-cooperative tumbling spacecraft, pose tracking, LiDAR, point cloud

## Abstract

How to determine the relative pose between the chaser spacecraft and the high-speed tumbling target spacecraft at close range, which is an essential step in space proximity missions, is very challenging. This paper proposes a LiDAR-based pose tracking method by fusing depth maps and point clouds. The key point is to estimate the roll angle variation in adjacent sensor data by using the line detection and matching in depth maps. The simplification of adaptive voxelized grid point cloud based on the real-time relative position is adapted in order to satisfy the real-time requirement in the approaching process. In addition, the Iterative Closest Point algorithm is used to align the simplified sparse point cloud with the known target model point cloud in order to obtain the relative pose. Numerical experiments, which simulate the typical tumbling motion of the target and the approaching process, are performed to demonstrate the method. The experimental results show that the method has capability of estimating the real-time 6-DOF relative pose and dealing with large pose variations.

## 1. Introduction

Pose estimation is one of the key technological challenges to enable relative navigation of a chaser spacecraft with respect to a target spacecraft. The relative pose is defined as a set of six parameters representing the translational and rotational degrees of freedom of the relative motion. The capability of the chaser spacecraft to accurately estimate its relative pose is important to guidance, navigation and control technologies for autonomous proximity and rendezvous in orbit, especially in close-range proximity. Most of the on-orbit defective spacecraft are non-cooperative objects, such as derelict or defective satellites. Therefore, the research on the relative pose determination of non-cooperative spacecraft is of great significance, which has become a hotspot and can be applied to many space missions like active debris removal, space manipulation, on-orbit servicing, etc. [1,2,3,4,5].

According to the specific mission scenario, a suitable pose determination technology includes the most suitable sensor and the effective pose estimating method. Usually monocular vision [6,7], stereo vision [8,9], light detection and ranging (LiDAR) sensors [5,10,11,12,13,14,15,16,17,18] and time-of-flight (ToF) depth cameras [19,20,21,22,23] are the common sensors used in close range tasks. The key point of the pose method largely depends on the nature of the target spacecraft and the characteristic of the specific sensor type.

Different from the cooperative target spacecraft, the non-cooperative target spacecraft usually cannot communicate with the chaser spacecraft, and there are not any artificial markers mounted on the body surface. The target usually revolves around its axes of maximum inertia due to the effects of various perturbation forces, which is typical of uncontrolled satellite. In particular, large scale changes in close-range proximity would greatly affect the measured result. Also, the real-time requirement of the pose estimation method is vital for the subsequent navigation and control operation. Hence, it is a challenging issue to accurately obtain the real-time 6-DOF relative pose between the chaser and the non-cooperative tumbling target spacecraft.

### 1.1. Related Works of LiDAR Based Relative Pose Determination

Many pose estimation studies have been conducted in recent years. The optical vision-based methods have been widely studied to enhance the relative pose estimation ability. The monocular camera is adopted to estimate the relative pose by solving the PNP problem [6,7]. It obviously lacks depth information. Stereo vision cameras are widely used, although their effective range is restricted to the baseline length between the two cameras. The Argon system [8], which is the typical visible stereo vision device to obtain the relative pose, has been developed for the SUMO/FREND project [9] and has been tested on STS-125 flight experiments.

Nowadays, the active range sensors such as scanning LiDAR, flash LiDAR, ToF camera, have gained a lot of attention in space relative navigation applications. LiDAR sensors are robust against lighting changes.

The scanning LiDAR can collect one point cloud data at a time. It cannot avoid the data distortion problem when observing a moving target. The TriDAR system [10], which uses the triangulation and scanning LiDAR technology to provide the 6-DOF relative pose, has been developed and tested on STS-128, STS-131, STS-135. A novel congruent tetrahedron align algorithm is presented to estimate the initial relative pose by using the sparse unorganized point cloud acquired by the scanning LiDAR [11].

Recently, the flash LiDAR and ToF camera have increasingly become popular for pose estimation. Unlike the scanning LiDAR, they can collect the entire point cloud data at once, and have the advantages of high frame rate, simple structure and high integration. Several flash LiDAR sensors have been tested during rendezvous with the International Space Station, such as Ball Corp’s flash LiDAR is tested on the STS-134, the ASC’s DragonEye flash LiDAR is selected by SpaceX and tested on the STS-127 and STS-133.

In close range proximity, flash LiDAR is more effective than scanning LiDAR, as it can avoid point cloud distortion when the non-cooperative spacecraft is rotating and translating. The output data of the ToF camera is similar as that of the flash LiDAR. ToF cameras are also promising for real-time space measurement [19,20,21,22,23] and have been used for ground tests.

Some researchers are focused on the topic of 3D point cloud-based non-cooperative spacecraft relative pose determination [12,13,14,15,16,17,18]. Usually, the non-cooperative targets can be divided into two categories, depending on whether the geometrical information about their shape and size is available, or they are fully unknown. For most of the on-orbit servicing missions, the target belongs to the former case as the target model is available. For pose initial acquisition, a 3D template matching technique is designed in [12], and the modal-based pose estimation method [13,14] is proposed. In [15], the Oriented, Unique, and Repeatable Clustered Viewpoint Feature Histograms (OUR-CVFH) method is proposed for pose initialization. A novel pose initial method is proposed in [16] by directly aligning the dense point cloud data with the known model. For pose tracking in sequence data, most methods directly adopt the iterative closest point (ICP) algorithm [24] or variants. As given in [11,13,14,15,16], the sensor point cloud data are directly used for ICP and the pose value is obtained when the ICP algorithm converges. Obviously, it is time-consuming when the number of the point cloud data is large. Also, when the target is tumbling rapidly, the ICP algorithm may not converge to the optimal value due to the large difference in adjacent sensor data. Besides, the feature points are firstly identified by using curvature, normal and other geometric features of the point cloud to reduce the sensor data number [17,18]. By fusing a ToF depth camera and a coupled high-resolution monocular sensor, multi-sensor solutions have been discussed to improve the effectiveness of the pose estimation [22,23]. However, the device is complex and the off-line camera calibration process is needed. For a full navigation solution, the filter scheme is also important in order to obtain a reliable and time-effective estimate of the relative pose. A dual-state inertial extended Kalman filter is designed and tested in two approach scenarios [15]. Based on the unscented Kalman filter, a novel filter scheme is proposed [25], which can estimate the shape, the relative attitude, position and velocity of a non-cooperative target by using the target’s attitude dynamics and the chaser’s relative orbital dynamics.

Up to now, the relative pose determination in close range is still an open research area, especially involving the non-cooperative target spacecraft. This case is very complex since the target spacecraft is free tumbling with a high roll speed. There are very few methods to discuss this case. Also, as the limited processing resource is available on board, the algorithm efficiency should be considered.

### 1.2. Objectives and Contributions

In this paper, we focus on the non-cooperative tumbling spacecraft pose tracking method in close-range proximity mission, such as close-range rendezvous and final approach. The flash LiDAR sensor is adopted in our method. The model data of the non-cooperative target is assumed to known, which means that the geometric model is available. Under this assumption, a LiDAR based spacecraft pose tracking method is designed in order to estimate the 6-DOF relative pose with balance the accuracy and efficiency. The main contributions of our work are:

- The consistence of the depth map and the point cloud is explored, and a depth map aided point cloud registration strategy is proposed to obtain high accuracy relative pose.
- The roll angle variation in adjacent sensor data is computed by detecting and matching the lines from the adjacent depth map.
- A point cloud simplification process based on the real-time relative position is designed to reduce the computing time.
- For approaching the tumbling non-cooperative target in close range, the simulated sensor data are generated and the numerical simulations are conducted.

The rest of this paper is arranged as follows: in Section 2, we describe the proposed relative pose estimation method in detail. The experimental results and the discussion are presented in Section 3. Conclusions are given in Section 4.

## 2. Proposed Relative Pose Estimation Method

In this section, the proposed relative pose estimation method is described in detail.

### 2.1. Definition of Reference Frames and Relative Pose Parameters

**Figure 1:**
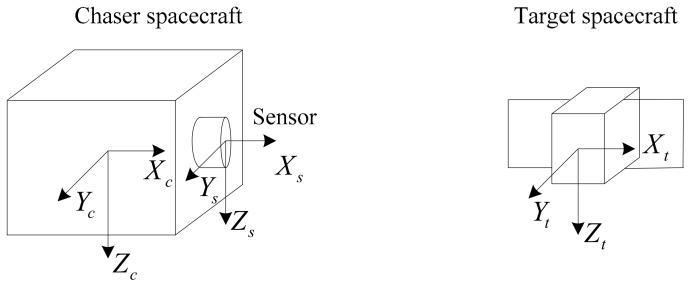
The definition of reference frames.

For the relative navigation applications in this paper, three reference frames are of interest: the chaser body-fixed frame $O_{c}-X_{c}Y_{c}Z_{c}$, the sensor frame $O_{s}-X_{s}Y_{s}Z_{s}$, and the target/modal frame $O_{t}-X_{t}Y_{t}Z_{t}$, as shown in Figure 1.

The origin of the frame $O_{c}-X_{c}Y_{c}Z_{c}$ and the frame $O_{t}-X_{t}Y_{t}Z_{t}$ separately lies in the mass center of the chaser spacecraft and the target spacecraft. The origin of the sensor frame $O_{s}-X_{s}Y_{s}Z_{s}$ lies in the optical center of the sensor. In this paper, we define that the axis $O_{c}X_{c}$ is pointing towards the target, and the axis $O_{c}Z_{c}$ points to the center of the earth and the axis $O_{c}Y_{c}$ obeys the right-hand role. The axes orientations of $O_{t}-X_{t}Y_{t}Z_{t}$ and $O_{s}-X_{s}Y_{s}Z_{s}$ are parallel with $O_{c}-X_{c}Y_{c}Z_{c}$. The reference frames may be oriented in a different way when required.

The transformation matrix from the sensor frame $O_{s}-X_{s}Y_{s}Z_{s}$ to the chaser body-fixed frame $O_{c}-X_{c}Y_{c}Z_{c}$ is known by the structural design. Thus we focus on the estimating the transformation matrix from the sensor frame $O_{s}-X_{s}Y_{s}Z_{s}$ to the target/modal frame $O_{t}-X_{t}Y_{t}Z_{t}$ by processing the flash LiDAR sensor data.

The definition of the six-DOF relative pose parameters is defined as Equations (1) and (2). The relative position, which represents the position of the chaser with respect to the target, is indicated as the vector T. The relative attitude, which aligns the sensor frame and the target/modal frame, is represented as the rotation matrix R by a roll-pitch-yaw sequence of Euler angles. Rotation about the X axis, Y axis and Z axis is represented in order by the roll angle φ, the pitch angle θ and the yaw angle ψ. So the transformation matrix H between the sensor frame and the target/modal frame can be expressed by R and T as Equation (3):

(1) $$T={\left[\Delta x,\Delta y,\Delta z\right]}^{T}$$

(2) $$R=\left[\begin{array}{ccc} \cos\psi \cos\theta & \sin\psi \cos\varphi -\cos\psi \sin\theta \sin\varphi & \sin\psi \sin\varphi -\cos\psi \cos\varphi \sin\theta \\ -\sin\psi \cos\theta & \cos\psi \cos\varphi -\sin\psi \sin\varphi \sin\theta & \cos\psi \sin\varphi -\sin\psi \sin\theta \cos\varphi \\ \sin\theta & -\cos\theta \sin\varphi & \cos\theta \cos\varphi \end{array}\right]$$

(3) $$H=\left[\begin{array}{cc} R & T\\ 0 & 1 \end{array}\right]$$

### 2.2. The Framework of the Proposed Method

**Figure 2:**
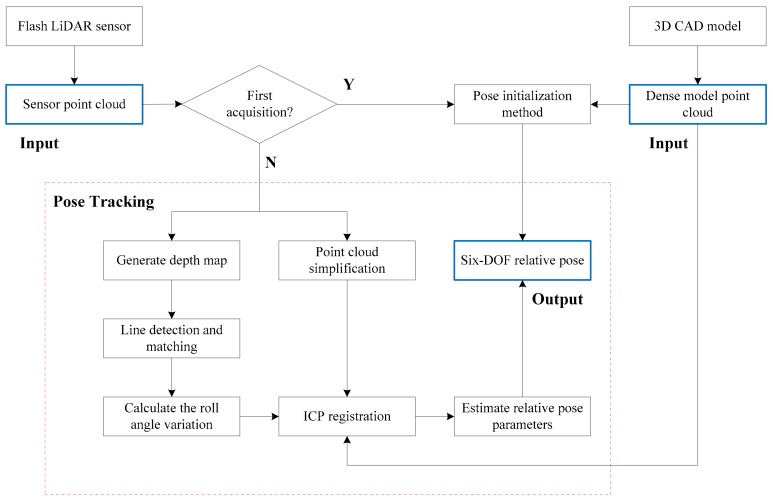
Framework overview.

The framework of the proposed relative pose tracking method is illustrated in Figure 2. The flash LiDAR data and the dense model point cloud data of the target are the input of the data processing, and the six-DOF relative pose parameters are the output, which can be used for the pose and orbit control system.

To accomplish a full relative pose solution, two main processes, which includes pose initialization and pose tracking, are required. Pose initialization is performed when the first sensor point cloud is acquired. Pose tracking is performed to generate the continuous pose output by using the sensor point cloud at a frame rate. In this work, we aim to present an effective and efficient pose tracking method which addresses the issues of approaching the tumbling non-cooperative target spacecraft in close-range. Several main steps are designed including generating the depth map, line detection and matching, calculate the roll angle variation, point cloud simplification and calculate the relative pose parameters. The detailed descriptions are given in the following sections.

For the model-known target spacecraft, the dense model point cloud can be obtained offline through the 3D CAD model of the target by finite element analysis. When dealing with the fully unknown target spacecraft, one solution is to build the modal directly on board using the sensor by the three-dimensional reconstruction technology [26] through the chaser flying around the target. This case is out of the scope of the paper.

### 2.3. Sensor Point Clouds and Depth Maps

The flash LiDAR sensor outputs the point cloud data $P_{s}={\left\{p_{s,i}\right\}}_{i=1:R\times C}$, which represents the coordinate of each point in the Cartesian coordinate system. $p_{s,i}\left(x_{i},y_{i},z_{i}\right)$ is defined as one point cloud data in the sensor frame $O_{s}-X_{s}Y_{s}Z_{s}$. The parameters of the flash LiDAR sensor include resolution R×C, which represents the number of rows and columns respectively, and field of view $\alpha_{v}\times \alpha_{h}$.

According to the definition of the frames in Section 2.1, we can convert the point cloud data $P_{s}$ to the depth map I. The resolution of the depth map I is also R×C, which is the identical as the resolution of the flash LiDAR sensor. The gray value of each pixel is computed as Equation (4):

(4) $$\{\begin{array}{c} I\left(r,c\right)=255\times \left[1-\left(x_{i}-x_{\min}\right)/\left(x_{\max}-x_{\min}\right)\right]\\ r=\arctan\left(z_{i}/x_{i}\right)/\left(\alpha_{v}/R\right)+R/2\\ c=\arctan\left(y_{i}/x_{i}\right)/\left(\alpha_{h}/C\right)+C/2 \end{array}$$

where the $x_{\max}$ and $x_{\min}$ are computed by using the point coordinate value in $O_{s}X_{s}$ axis of all the points for each sensor point cloud. The r is the index in rows which corresponds to the axis $O_{s}Z_{s}$ and the c is the index in columns which corresponds to the axis $O_{s}Y_{s}$. The range of gray value in the depth map I is [0,255].

**Figure 3:**
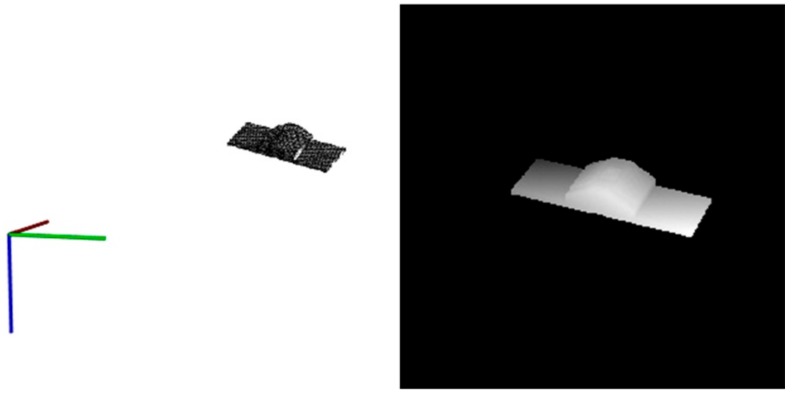
The point cloud data in 3D view (**left**) and the converted depth map (**right**).

An example of the conversion process is shown in Figure 3. The point cloud data is shown in 3D view in left and the corresponding depth map is shown in right. The axis in left represents the view position of the sensor. We can see that the boundary of the spacecraft is clear in depth map due to the high precision laser measurement. So the boundary feature information in depth image can be used to aid the pose processing.

### 2.4. Line Detection and Matching

When the chaser spacecraft approaches the target spacecraft in close range, we assume that the body of the target spacecraft always lies in the sensor’s field of view. Rather than the point feature detection and description, the line feature is more robust for human-made spacecraft in space environment. It is difficult to detect the line feature of the target spacecraft in 3D point cloud P, so the depth map I is adapted by using the mature 2D line feature detection method.

In this paper, the Line Segment Detector (LSD) algorithm [27] is used to detect the line feature of the depth map I. Its advantages are no parameter tuning, low computational burden and low error rate in the complex image. The main process steps include the image gradient computing, the region growing algorithm, the rectangular approximation of regions and line segment validation. The details can be seen in [27].

To obtain the accurate line feature of the boundary, a preprocessing downsampling step is combined with the LSD algorithm, as Equations (5) and (6). This preprocess can smooth irrelevant details of the depth image in order to avoid getting many short lines, also, it can reduce the computational time of the line detection by downsampling the original depth image:

(5) $$I_{s}=G_{d}\left(I\right)$$

(6) $$L=\left\{l_{i}\right\}=LSD\left(I_{s}\right)$$

where $G_{d}$ is a Gaussian downsampling function. The Gaussian kernel is 5×5, the standard deviation of the Gaussian is set to 1 and the downsampling factor is set to 2 as an empirical value. $I_{s}$ is the Gaussian blurred downsampling version of the depth map I. LSD represents the LSD line detection algorithm. L is the collection of the LSD detection results and $l_{i}$ is the *i*-th line.

Line matching is used to obtain the roll angle variation in adjacent sensor data. So the line feature descriptors and the matching criterion should be defined. In this paper, for each line, we compute its four feature value to establish the line descriptors, including the line length, line gradient magnitude, line orientation angle, and line middle point position.

For the *i*-th line $l_{i}$, the definition of the line length $len\left(l_{i}\right)$, line orientation angle $\phi \left(l_{i}\right)$, line middle point position $\left(m_{xi},m_{yi}\right)$ is obvious. The line gradient magnitude $gm\left(l_{i}\right)$ is defined as Equation (7):

(7) $$gm\left(l_{i}\right)=\frac{1}{N}\sum_{n=1}^{N}gm\left(p_{n}\right)$$

where N is point number of the line $l_{i}$, $p_{n}$ is one point of the line $l_{i}$, $gm\left(p_{n}\right)$ is the gradient magnitude of point $p_{n}$, so gm represents the average gradient magnitude of the line $l_{i}$.

We define the similarity degree of the line descriptors separately as the Equations (8)–(11). In order to describe concisely, we use the subscript a and b to represent two different lines. The value range of $sm_{1}$,$sm_{2}$,$sm_{3}$,$sm_{4}$ is [0,1]:

(8) $$sm_{1}\left(l_{a},l_{b}\right)=\min\left(len\left(l_{a}\right),len\left(l_{b}\right)\right)/\max\left(len\left(l_{a}\right),len\left(l_{b}\right)\right)$$

(9) $$sm_{2}\left(l_{a},l_{b}\right)=\min\left(gm\left(l_{a}\right),gm\left(l_{b}\right)\right)/\max\left(gm\left(l_{a}\right),gm\left(l_{b}\right)\right)$$

(10) $$sm_{3}\left(l_{a},l_{b}\right)=\cos\left(\phi \left(l_{a}\right)-\phi \left(l_{b}\right)\right)$$

(11) $$sm_{4}\left(l_{a},l_{b}\right)=\frac{1}{2}\left(\frac{\min\left(m_{xa},m_{xb}\right)}{\max\left(m_{xa},m_{xb}\right)}+\frac{\min\left(m_{ya},m_{yb}\right)}{\max\left(m_{ya},m_{yb}\right)}\right)$$

The four similarity metrics can evaluate the similarity degree of the lines in different aspects in order to obtain the correct matched line pair. For the axes symmetrical uncooperative spacecraft, the single similarity metric, such as the line orientation angle, is difficult to distinguish the two symmetry boundary lines of the axes symmetrical satellite. Besides, it may be difficult to distinguish the boundary line from the inner line when only using the line gradient magnitude.

Thus, the matching criterion is defined by weighting the four similarity degree as Equation (12):

(12) $$sm_{a,b}=\alpha \times sm_{1}\left(l_{a},l_{b}\right)+\alpha \times sm_{2}\left(l_{a},l_{b}\right)+\alpha \times sm_{3}\left(l_{a},l_{b}\right)+\alpha \times sm_{4}\left(l_{a},l_{b}\right)$$

where α is the fixed weighting factor and it is set to 0.25. The value range of $sm_{a,b}$ is [0,1].

The above equation shows that the larger the $sm_{a,b}$ value, the more similar the two lines. The lines are detected separately by using the previous (*k*−1)-th frame data and the current *k-*th frame data. Base on this matching criterion, the line similarity degree matrix in adjacent sensor data can be calculated.

### 2.5. The Roll Angle Variation Calculation

The angular variation of the same line in adjacent depth map contains the information of the global rotation transformation. As a result, the roll angle variation in adjacent sensor data can be obtained by matching the lines in the (*k*−1)-th frame data and the current *k*-th frame data.

Usually, the number of the lines varies in each frame data. Thus, after detecting and describting the lines in the *k-*th depth map, we first sort the lines by the line length $len\left(l_{i}\right)$ in descending order. Then we take the first few lines to compute the line similarity degree matrix. Define Q is the number of the selected lines, so we set $Q_{k-1}$ and $Q_{k}$ as the number of the selected lines in the (*k*−1)-th depth map and the *k-*th depth map, respectively.

Let $L_{k-1}={\left\{l_{i}\right\}}_{i=1:Q_{k-1}}$ and $L_{k}={\left\{l_{j}\right\}}_{j=1:Q_{k}}$ are the lines collection of the (*k*−1)-th frame and the *k*-th frame respectively.

The line similarity degree matrix SDM is defined as Equation (13):

(13) $$SDM=\left[\begin{array}{cccc} sm_{1,1} & sm_{1,2} & \cdots & sm_{1,j}\\ sm_{2,1} & \cdots & \cdots & \vdots \\ \vdots & \cdots & \cdots & \vdots \\ sm_{i,1} & \cdots & \cdots & sm_{i,j} \end{array}\right]$$

where $sm_{i,j}$ is calculated as Equation (12). The subscript i is the index of the lines in $L_{k-1}$, and the subscript j is the index of the lines in $L_{k}$.

For each column, we compute the maximum value, whose subscripts indicate the corresponding lines index. To reduce the possibility of the mismatch, the threshold $T_{d}$ is adopted and is set to 0.8 as an empirical value. For each column, when the maximum value is larger than the threshold $T_{d}$, the correct matched line pair is obtained. So we can obtain the correct matched line corresponding pairs in $L_{k-1}$ and $L_{k}$.

We calculate the roll angle variation Δϕ by averaging the line orientation angle variation of all the line corresponding pair, as in the Equation (14):

(14) $$\Delta \phi =\frac{1}{Q_{m}}\sum_{i=1}^{Q_{m}}\left(\phi \left(l_{a}\right)-\phi \left(l_{b}\right)\right)$$

where $Q_{m}$ is the number of the correct matched line pairs.

According to the roll angle variation value Δϕ and the definition of reference frames in Figure 1, the transformation matrix $H_{roll}$ can be expressed in Equation (15), which reflects the roll angle variation information between the (*k*−1)-th frame data and the current *k*-th frame data.

(15) $$H_{roll}=\left[\begin{array}{cccc} 1 & 0 & 0 & 0\\ 0 & \cos\Delta \phi & \sin\Delta \phi & 0\\ 0 & -\sin\Delta \phi & \cos\Delta \phi & 0\\ 0 & 0 & 0 & 1 \end{array}\right]$$

**Figure 4:**
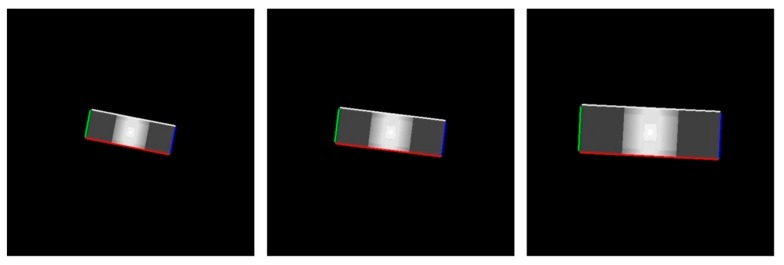
The example of the line matching result in depth map (from left to right: partial results of the sensor sequence data).

An example of the line matching result in the adjacent depth map is shown in Figure 4. We can see that the boundary of the target spacecraft can be detected and the corresponding lines, which are shown with the same color, are correctly matched while the target spinning motion and the scale changes exist in sensor sequence data.

### 2.6. Point Cloud Simplification

The flash LiDAR can capture the dense point cloud data with fixed resolution and Field of View. When the chaser spacecraft is approaching the target spacecraft, the smaller the relative distance, the larger the target in the FOV of the sensor occupies. It is obvious that the number of the points in the sensor point cloud would rapidly increase. This case will cause the increase of the executing time for most of the ICP registration algorithm. In an extreme case, the non-real-time relative pose result may lead to the destabilization of the pose and orbital control system.

How to deal with the dense sensor point cloud is a key issue. Hence, point cloud simplification is needed. Many scholars have made great success on the point cloud simplification method, such as k-means clustering based method, normal and curvature based method et al. [28]. The voxelized grid simplification method is a start-of-the-art method and can be efficiently executed by the open source Point Cloud Library (PCL) [29].

The challenge of reduce the point number of the sensor point cloud and get a stable ICP execution time, is that how to reduce the point number to a constant level in the approaching process. In the paper, an adaptive parameter strategy is designed. The grid size, which is used in the voxelized grid simplification method as the unique parameter, is computed adaptively according to thereal-time relative position.

The grid size $d_{k}$ in the *k*-th sensor data is calculated according to the real-time relative position in the axis $O_{s}X_{s}$ as Equation (16). The grid size is adaptive for each sensor point cloud data in the approaching process:

(16) $$d_{k}=d_{m}\times \exp\left(\frac{X_{init}-X_{k-1}}{X_{init}}\right)$$

where $d_{m}$ is the size of the model point cloud grid. $X_{init}$ is the initial relative distance, which can be obtained by the pose initialization method. $X_{k-1}$ is the real-time relative position value computed by the proposed pose tracking method in the (*k*−1)-th sensor data.

**Figure 5:**
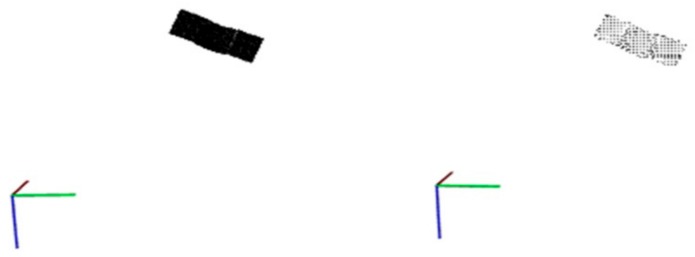
The example of the point cloud simplification (**left**: the dense point cloud data; **right**: the result of the simplification process).

An example of the point cloud simplification is shown in 3D view in Figure 5. The relative distance is 20 m. In Figure 5, the axis represents the view position of the sensor. The dense point cloud in left is the output of the sensor, which is generated by the sensor simulator. The sparse point cloud in right is the result of the simplification process. The number of points in the dense point cloud is about 20,000, and the number of points in the sparse point cloud is about 4500.

This point cloud simplification process can efficiently obtain the sparse sensor point cloud data with a simple procedure. Thus, the point cloud registration process, which aligns the sensor point cloud data with the target modal point cloud data, would become significantly faster and easily carried out.

### 2.7. The Relative Pose Computation

Assume that the model point cloud of the target spacecraft is known, we can execute the pose tracking process to generate the continuous relative pose by aligning the model point cloud with the sensor point cloud at a frame rate.

The model point cloud is $P_{m}$. The current *k*-th sensor point cloud is $P_{s}^{k}$. The previous transformation matrix is defined as $H_{k-1}$. The detail process is depicted as follows:

Firstly, the matrix $H_{roll}$ is calculated by the process in Section 2.3, Section 2.4 and Section 2.5. The $P_{s}^{k}$ is simplified by the process in Section 2.6 and the sparse sensor point cloud is obtained.

Secondly, we transform sparse sensor point cloud by the matrix $H_{k-1}$ and the matrix $H_{roll}$ in sequence. Then the converted sensor point cloud is aligned with the model point cloud $P_{m}$ by using the point-to-point ICP algorithm. The transform matrix $H_{icp}$, which is the result of the ICP, can be obtained when the ICP algorithm converges. Thus, the current *k*-th transformation matrix can be repressed as the Equation (17):

(17) $$H_{k}=H_{icp}H_{roll}H_{k-1}$$

So the 6-DOF relative pose parameters can be calculated from the $H_{k}$ according to the Equations (1)–(3). For each new sensor measurement, the same process is conducted and the pose tracking is realized. Especially, for the first sensor data, the transformation matrix $H_{1}$ is calculated by pose initialization method.

Specifically, in this work, the parameters of the ICP algorithm are the ICP error threshold and the maximum number of iterations. As an empirical value, the ICP error threshold is 10^−6^ m^2^, which measures the variation of the ICP error among two subsequent iterations. The maximum number of iterations is 100, which prevents the ICP algorithm from taking too long. The ICP algorithm converges when the ICP error becomes less than the given threshold or the max iteration number is reached. The whole procedure of the proposed pose tracking method is summarized in Algorithm 1.
**Algorithm 1:** The Procedure of the Proposed Pose Tracking Method**Input**: The model point cloud $P_{m}$, the current *k*-th sensor point cloud $P_{s}^{k}$ While k>1 1: Convert the $P_{s}^{k}$ to the depth map I. 2: Detect the lines collection L in the depth map I. 3: Calculate the lines descriptors for each line. 4: Sort the lines by the line length len in descending order. 5: Select the first $Q_{c}$ lines and calculate the line similarity degree matrix SDM. 6: Calculate the roll angle variation Δϕ and the transformation matrix $H_{roll}$. 7: Perform the point cloud simplification process for $P_{s}^{k}$ and obtain the sparse sensor point cloud. 8: Calculate the transform matrix $H_{icp}$ by aligning the sparse sensor point cloud with the $P_{m}$. 9: Calculate the current *k*-th transformation matrix $H_{k}$. 10: Calculate the six-DOF relative pose parameters. 11: k=k+1, and go to step 1. end **Output**: the six-DOF relative pose, including the roll angle φ, the pitch angle θ, the yaw angle ψ, the Δx, the Δy, the Δz.

## 3. Experiments

### 3.1. Test Setup

**Figure 6:**
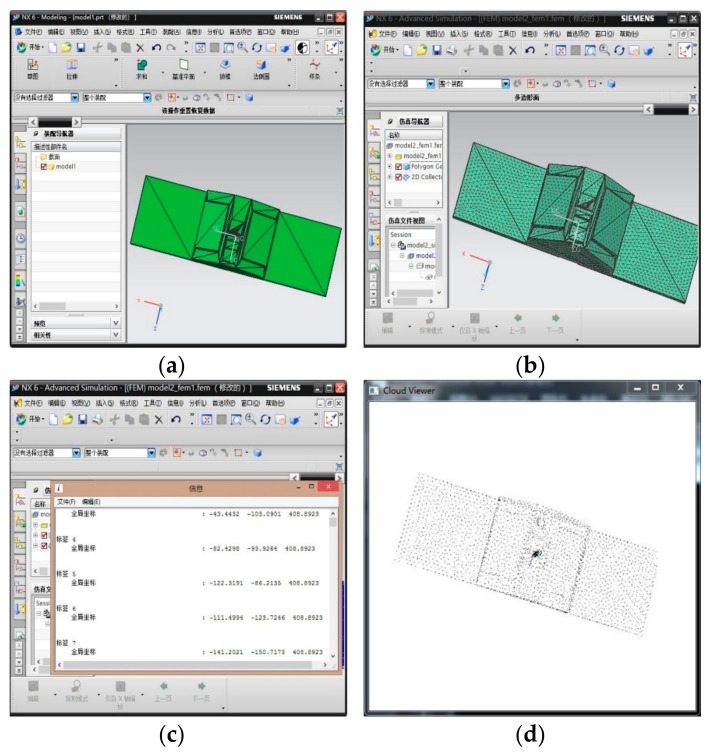
The procedure of generating the model point cloud from the 3D CAD target model (**a**) the CAD model is loaded in the UG software; (**b**) the result of finite element analysis; (**c**) the output of the point coordinate of the triangular mesh; (**d**) the 3D display of the model point cloud.

To test the performance of the proposed method, a set of emulational experiments were conducted. The Cyclone Global Navigation Satellite System (CYGNSS) satellite is adopted as the target spacecraft in the experiments. The 3D model of the CYGNSS satellite can be obtained from the corresponding public dataset [30]. For experiment convenience, the model size in use is enlarged to 3550 mm × 1100 mm × 470 mm, so the model point cloud can be generated through the 3D CAD model of the target by finite element analysis software. In this paper, the UG software is used and the size of mesh grids is set to 50 mm, which is also considered as the spatial resolution of the model point cloud. The procedure of generating the model point cloud from the 3D CAD target model is given in Figure 6.

The flash LiDAR is adopted as the sensor. The parameters of the sensor include resolution, field of view and the maximum absolute error, which usually are fixed to a certain type of sensor and given in the product datasheet. In the paper, the resolution is set to 500 (horizontal) × 500 (vertical), the field of view is set to 20° (horizontal) × 20° (vertical) and the maximum absolute error is set to 10 mm, which represents the range bias and the range noise.

In order to generate the sensor point cloud and the true relative pose value, we programmed a sensor point cloud simulation software according to the principal of the flash LiDAR. Supposing that the model point cloud is available, the main steps of generating the sensor point cloud are depicted as follows:

*Step 1*: according to the known observed position and attitude, the model point cloud $P_{m}$ is transformed to the sensor frame $O_{s}-X_{s}Y_{s}Z_{s}$. Also, the given transformation parameters represent the true relative pose value.

*Step 2*: according to the sensor resolution and field of view, for one laser light, a point $p_{t,i}\left(x_{i},y_{i},z_{i}\right)$ can be obtained by calculating the intersection point of the laser straight light and the triangular mesh of the model surface.

*Step 3*: Considering that the ranging bias and noisy exist, a random distance error Δd is added as the Equation (18) and get the corresponding sensor point $p_{s,i}\left(x_{i},y_{i},z_{i}\right)$:

(18) $${\left\|p_{t,i}\right\|}^{2}+\Delta d={\left\|p_{s,i}\right\|}^{2}$$

where Δd is set to a random value in the range $\left[-\Delta d_{\max},\Delta d_{\max}\right]$. $\Delta d_{\max}$ is the maximum absolute error.

*Step 4*: For each laser light of the Flash LiDAR sensor, the corresponding sensor point is calculated by performing the Step 2 and Step 3. So the sensor point cloud data $P_{s}$ can be generated.

According to the preset relative motion conditions, the sequence sensor point cloud data can be generated based on the above steps by changing the given observed position and attitude.

The sensor point cloud simulation software is implemented with C++ codes and used in the experiments. Other point cloud simulation software can also be used such as the reference [31].

Most of the existing model-based pose estimation methods, such as [11,13,14,15,16], directly use the ICP method to calculate the relative pose in the pose tracking phase, so in this paper, we compare the proposed pose tracking method (denoted as “Proposed”) with the typical point to point ICP method (denoted as “ICP”).

To accomplish a full relative pose estimation process, the pose initialization method is achieved by the global optimal searching based method, which is our previous work [16]. The first sensor point cloud is used for pose initialization. The compared “ICP” method uses the first sensor point cloud directly, the “Proposed” method performs the line detection and the point cloud simplification on the first sensor point cloud before performing the pose initialization method.

We evaluate the two pose tracking method in the aspects of accuracy and computational efficiency in the following experiments. The relative pose error in 6-DOF is computed respectively, which is defined as the difference between the truth value and the result of the pose tracking method. The runtime of the compared “ICP” method is defined as the ICP registration time because the compared “ICP” method aligns the sensor point cloud with the model point cloud directly, no pre-processing is performed. The runtime of the “Proposed” method consists of the processing time of all the processing steps. The CPU is a dual core 2.9 GHz and the RAM is 3 GB. The code is implemented with C++ and PCL.

The tumbling motion of the non-cooperative spacecraft can appear due to the perturbation torque on orbit involving solar pressure, gravitational gradient and the residual angular momentum. There are usually two typical cases needed to be analyzed [32]. One is that the target spacecraft is spinning rapidly around only one axis, which is generally the maximum or minimum inertia axis. The other one is that the target spacecraft has a complex spinning motion with a nutation angle. Based on the analysis of the tumbling motion, the numerical simulation experiments are designed in order to evaluate the pose tracking performance in the two cases respectively.

### 3.2. Emulational Experiment 1

To test the proposed pose tracking method in the simple spinning motion case, the experiment is carried out under the following simulated conditions. Suppose the target spacecraft is rotating only on *X* axis with a constant angular velocity, which means the relative roll angle is time-varying. The chaser is in rendezvous motion with uniform velocity to the target.

The initial observed position (x,y,z) is set to (60, 0, 0), the units are in meters. The initial rotation (φ,θ,ψ) is set to (125, 0, 0), the units are in degrees. For each simulated sensor point cloud data, the φ is changing from 125° to −125° at 5° interval and the x distance is changing from 60 m to 10 m at 1 m interval. So the number of the simulated sensor point cloud data is 51.

**Figure 7:**
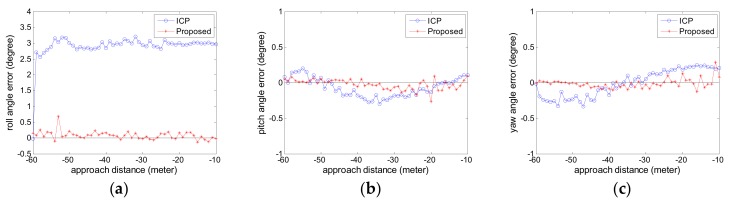
The attitude error curve in Experiment 1 (**a**) roll angle error; (**b**) pitch angle error; (**c**) yaw angle error.

**Figure 8:**
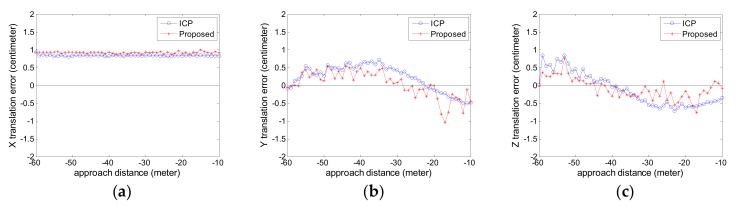
The position error curve in Experiment 1 (**a**) X translation error; (**b**) Y translation error; (**c**) Z translation error.

The estimated relative pose error is computed. The attitude error curves are given in Figure 7 and the position error curves are given in Figure 8. The negative sign about approach distance indicates that the chaser spacecraft is moving towards the target spacecraft along the *X* axis.

From the Figure 7 and Figure 8, we can see that the proposed pose tracking method can compute the accurate relative pose while the target spacecraft is spinning with a high roll speed. The rotation error is less than 0.5° and the position error is about 1 cm. The error varies slightly in the whole approaching movement process. Compared with the ICP method, the proposed method can obtain more precise relative pose values. This is particularly obvious on the roll angle in Figure 7a. For the other relative angle and the relative position, the two methods have similar high accuracy and the proposed method show slightly better performance than the ICP method.

**Figure 9:**
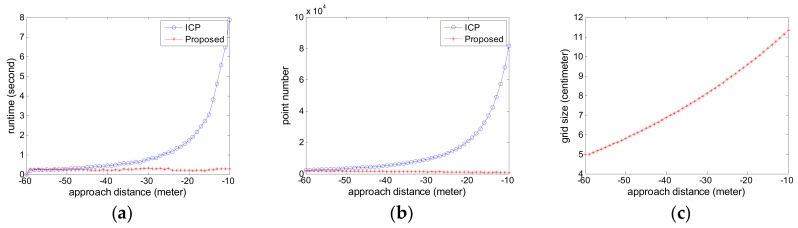
The runtime comparison in Experiment 1 (**a**) the runtime for each sensor data; (**b**) the point number of each sensor data; (**c**) the grid size used in the simplification process for each sensor data.

Figure 9a is the runtime for each sensor data in Experiment 1. Figure 9b shows the point number comparison of each original sensor point cloud data and the simplified point cloud data. Figure 9c shows the grid size used in the simplification process for each sensor point cloud data.

The ICP method in comparison uses the original sensor point cloud data while the proposed method adapts the adaptive voxelized grid simplification. As can be seen from the Figure 9a,b, the advantage of the proposed method is obvious. As the approaching distance decreases, the point number of the simulated sensor point cloud data increases sharply. For the compared ICP method, this case leads to an increase of the computational burden for the compared ICP method. Especially, when the approaching distance is less than 30 m, the runtime exceeds 1 s. This is usually unacceptable for the follow-up navigation and control operation. However, the proposed method can cope with the case. The point number of the simplified point cloud data is about 5000 at an arbitrary distance. The runtime of the proposed method is about 200 ms for each sensor data, which corresponds to roughly 5 FPS. As can be seen from Figure 9c, the approaching distance decreases, the grid size increases, so the point number is reduced as shown in Figure 9b and the stable runtime in the approaching process is achieved as shown in Figure 9a.

### 3.3. Emulational Experiment 2

Another experiment is carried out to test the proposed method in the complex spinning motion with a nutation angle. The simulated conditions are as follows. Suppose the target spacecraft is rotating on *X* axis with a constant angular velocity, which means the relative roll angle is time-varying. Meanwhile, the target spacecraft is rotating on *Y* axis with a time-varying nutation angle. The chaser is in rendezvous motion with uniform velocity to the target and the relative motion exists on the *X* axis and *Y* axis simultaneously.

The initial observed position (x,y,z) is set to (60, 10, 0), the units are in meters. The initial rotation (φ,θ,ψ) is set to (125, 10, 0), the units are in degrees. For each simulated sensor point cloud data, the φ is changing from 125° to −125° at 5° interval, the θ is changing from 10° to 0° at 0.2° interval, the x distance is changing from 60 m to 10 m at 1 m interval, and the y distance is changing from 10 m to 0 m at 0.2 m interval, so the number of the simulated sensor point cloud data is 51.

Different from Experiment 1, suppose the target spacecraft is free tumbling with a nutation angle, the attitude and the position change along *X* axis and *Y* axis for each simulated sensor data.

**Figure 10:**
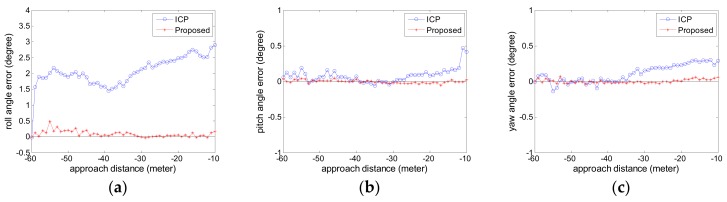
The attitude error curve in Experiment 2 (**a**) roll angle error; (**b**) pitch angle error; (**c**) yaw angle error.

**Figure 11:**
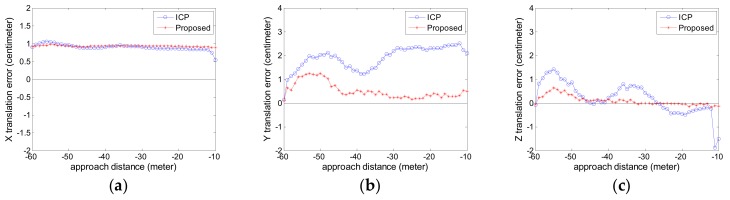
The position error curve in Experiment 2 (**a**) X translation error; (**b**) Y translation error; (**c**) Z translation error.

The estimated relative pose error is calculated. The attitude error curves are given in Figure 10 and the position error curves are given in Figure 11. The negative sign about approach distance indicates that the chaser spacecraft is moving towards the target spacecraft along the *X* axis.

From the Figure 10 and Figure 11, we can see that the proposed pose tracking method can deal with the complex target motion case and compute the accurate relative pose while the target spacecraft is spinning rapidly with a nutation angle. The rotation error is less than 0.5° and the translation error is about 1 cm. The proposed method can obtain more precise relative attitude values than the compared ICP method, especially on the roll angle shown in Figure 10a. In Figure 11, the two methods show high precision especially on the *X* axis, which is vital for avoiding the risk of collision. The proposed method can obtain more precise relative position value, especially on the *Y* axis shown in Figure 11b.

**Figure 12:**
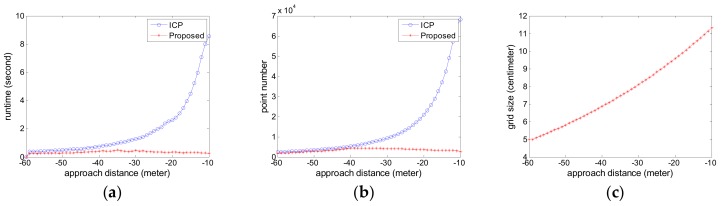
The runtime comparison in Experiment 2 (**a**) the runtime for each sensor data; (**b**) the point number of each sensor data; (**c**) the grid size used in the simplification process for each sensor data.

We also analyze the runtime and the point number comparison for each sensor data, shown in Figure 12a,b. Figure 12c shows the grid size used in the simplification process for each sensor point cloud data.

For the ICP method in comparison, the point number and the runtime increase rapidly as the approach distance decreases, especially when the approach distance is less than 30 m. However, the proposed method can obtain the better tradeoff between the precision and runtime than the compared ICP method. As can be seen from Figure 12a, the runtime of the proposed method is about 200 ms for each sensor data, which corresponds to roughly 5FPS. As can be seen from Figure 12b, the point number of the simplified point cloud data is about 5000 at an arbitrary distance. As can be seen from Figure 12c, the approaching distance decreases, the grid size increases. So the point number is reduced as shown in Figure 12b and the stable runtime in the approaching process is achieved as shown in Figure 12a. The grid size curve in Experiment 2 as shown in Figure 12c is slightly different from the curve in Experiment 1 as shown in Figure 9c. The reason is that the grid size is calculated with the Equation (16) according to the real-time relative position.

### 3.4. Discussion

The numerical simulation experiments and results are presented in Section 3.2 and Section 3.3 . From the Experiment 1 and the Experiment 2, we can see that the proposed pose tracking method can effectively estimate the real-time 6-DOF relative pose while the tumbling motion of the non-cooperative target spacecraft exists.

In the above experiments, the attitude error is less than 0.5°, and the position error is less than 1 cm. The results are more accurate than the results using the ICP based pose estimation method. The reason is that the proposed roll angle variation calculation strategy based on the line detection and matching in the adjacent depth map is effective, which can provide a good initial value for the point to point registration. Besides, the proposed method has high computational efficiency. It can satisfy the real-time requirement of the navigation and control system in the space applications.

The flash LiDAR sensor is a promising technology in space relative navigation tasks due to its unique advantages such as high frame rate, low mass and robustness. We demonstrate its usage on the relative pose determination in close range. In addition, the method discussed in this paper is also suitable for the ToF depth camera within its effective working range.

The numerical simulations can evaluate the performance of the pose determination methods. Also, it can test the performance of the specific sensor prior to field experiments. Extensive numerical simulation can be carried out through setting various simulation condition parameters. Because the straight line feature is common for most of the human-made spacecraft, the proposed method may be widely used. In future work, the robustness of the proposed method will be analyzed considering the various typical shapes of the targets.

## 4. Conclusions

In this paper, a high-accuracy and high-efficiency relative pose tracking method at close range is proposed for tumbling non-cooperative spacecraft. Based on the known target model and the flash LiDAR sensor data, a series of strategies are designed to achieve 6-DOF relative pose determination including performing the line detection and matching based on the converted depth map, calculating the roll angle variation, and adaptive point cloud simplification. The numerical simulation experiments, which simulate the scenarios of approaching the high-speed tumbling uncontrolled non-cooperative target, are conducted. In comparison with the standard ICP based pose tracking, the proposed method can estimate the 6-DOF relative pose with higher accuracy and computational efficiency and it can deal with the rapid pose changes effectively.

In future plan, some works will be concentrated on these aspects: (1) the targets with various shapes will be adapted to test the applicability of the method; (2) the field experiment also needs to be conducted with the hardware-in-the-loop system, which consists of the specific sensor, the mock-up of a specific target, and robotic arm based test platform et al.
